# Effect of roflumilast on inflammatory cells in the lungs of cigarette smoke-exposed mice

**DOI:** 10.1186/1471-2466-8-17

**Published:** 2008-08-28

**Authors:** Piero A Martorana, Benedetta Lunghi, Monica Lucattelli, Giovanna De Cunto, Rolf Beume, Giuseppe Lungarella

**Affiliations:** 1 Department of Physiopathology and Experimental Medicine, University of Siena, Siena, Italy; 2 Nycomed GmbH, Konstanz, Germany

## Abstract

**Background:**

We reported that roflumilast, a phosphodiesterase 4 inhibitor, given orally at 5 mg/kg to mice prevented the development of emphysema in a chronic model of cigarette smoke exposure, while at 1 mg/kg was ineffective. Here we investigated the effects of roflumilast on the volume density (V_V_) of the inflammatory cells present in the lungs after chronic cigarette smoke exposure.

**Methods:**

Slides were obtained from blocks of the previous study and V_V _was assessed immunohistochemically and by point counting using a grid with 48 points, a 20× objective and a computer screen for a final magnification of 580×. Neutrophils were marked with myeloperoxidase antibody, macrophages with Mac-3, dendritic cells with fascin, B-lymphocytes with B220, CD4+ T-cells with CD4+ antibody, and CD8+T-cells with CD8-α. The significance of the differences was calculated using one-way analysis of variance.

**Results:**

Chronic smoke exposure increased neutrophil V_V _by 97%, macrophage by 107%, dendritic cell by 217%, B-lymphocyte by 436%, CD4+ by 524%, and CD8+ by 417%. The higher dose of roflumilast prevented the increase in neutrophil V_V _by 78%, macrophage by 82%, dendritic cell by 48%, B-lymphocyte by 100%, CD4+ by 98% and CD8+ V_V _by 88%. The lower dose of roflumilast did not prevent the increase in neutrophil, macrophage and B-cell V_V _but prevented dendritic cells by 42%, CD4+ by 55%, and CD8+ by 91%.

**Conclusion:**

These results indicate (*i*) chronic exposure to cigarette smoke in mice results in a significant recruitment into the lung of inflammatory cells of both the innate and adaptive immune system; (*ii*) roflumilast at the higher dose exerts a protective effect against the recruitment of all these cells and at the lower dose against the recruitment of dendritic cells and T-lymphocytes; (*iii*) these findings underline the role of innate immunity in the development of pulmonary emphysema and (*iiii*) support previous results indicating that the inflammatory cells of the adaptive immune system do not play a central role in the development of cigarette smoke induced emphysema in mice.

## Background

Recently, chronic obstructive pulmonary disease (COPD) has been defined by the Global Initiative for Chronic Obstructive Lung Disease (GOLD) as a disease characterized by progressive, not fully reversible, flow limitation and "associated with an abnormal inflammatory response of the lungs to noxious particles and gases" [1]. Thus, a central role has been attributed to the chronic inflammatory response that in humans is present throughout the airways and parenchyma and that participates in the progression and exacerbation of this disease [2].

The attempt to reduce, with the use of anti-inflammatory agents, lung inflammatory cell infiltration is most appealing since such an effect would also reduce the lung burden of both proteases and oxidants. In an approach aiming at modulating the chronic inflammatory response, corticosteroids are used. However, these drugs have been found to be largely ineffective in attenuating inflammation in patients with COPD [3]. The resistance to corticosteroids may involve an impaired activity of the enzyme histone deacetylase, related to oxidative stress. In fact, a blunted activity of this enzyme is associated with a reduced response to corticosteroids and an enhanced expression of inflammatory cytokines [4].

Thus, as indicated in the GOLD guidelines, there is a pressing need to develop new agents capable of suppressing the inflammatory response [1]. The phosphodiesterases are a large family of intracellular enzymes that degrade cyclic nucleotides. Among these the phosphodiesterase 4 (PDE4) isoenzyme specifically targets 3', 5'-cyclic adenosine monophosphate (cAMP), a second messenger that exerts inhibitory effects on many inflammatory cells. Neutrophils, macrophages and CD8+ T-lymphocytes play a significant role in COPD and these cells have been shown to substantially express PDE4. Thus, substances that prevent the degradation of cAMP by inhibiting the activity of PDE4 will enhance the anti-inflammatory action of this second messenger [5].

In a previous investigation we reported that the specific PDE4 inhibitor roflumilast, given orally either at 1 mg/kg or at 5 mg/kg to mice acutely exposed to cigarette smoke, partially but significantly prevented the smoke-induced lung neutrophilia.

Additionally, when given orally at the same doses, in a chronic (7 months) model of cigarette smoke exposure, the high (5 mg/kg) but not the low dose of roflumilast completely prevented the smoke-induced development of emphysema and the drop in desmosine lung content. Thus, this study showed for the first time that a phosphodiesterase 4 inhibitor such as roflumilast, could fully prevent parenchymal destruction induced by cigarette smoke [6].

Recent reports indicate that inflammatory cells both of the innate (macrophages, neutrophils) and adaptive immune system (B lymphocytes, CD4+ T-lymphocytes, CD8+ T-lymphocytes) or cells linking innate and adaptive immunity (dendritic cells) may play an important role in the development of cigarette smoke-induced emphysema [7-9]. It was thus of interest to investigate the effect of roflumilast on the influx into the lung of the inflammatory cells of the innate and adaptive immune system. This was done with immunohistochemical methods coupled with a morphometrical assessment using the paraffin embedded blocks of the previous study.

This investigation could be of help in clarifying the importance of these cells for the development of cigarette smoke-induced emphysema. In fact, since the low dose of roflumilast did not prevent the development of emphysema while the high dose completely blocked it [6], it would be of interest to analyze the recruitment of inflammatory cells under both these conditions.

## Methods

In the present investigation the paraffin blocks of a previous study were used [6]. Briefly, five groups of 2 months old male mice of the strain C57 Bl/6J were treated as follows: 1) no treatment/air exposed, 2) roflumilast 5 m/kg p.o./air exposed, 3) no treatment/smoke exposed, 4) roflumilast 1 mg/kg p.o./smoke exposed, and 5) roflumilast 5 mg/kg p.o./smoke exposed. All animal experiments were conducted in conformity with the "Guiding Principles for Research Involving Animals and Human Beings" and approved by the Local Ethics Committee of the University of Siena.

After chronic exposure to room air or cigarette smoke for seven months, the animals were sacrificed and the lungs fixed in formalin (5%) at a pressure of 20 cm H_2_O. Lung volume was measured by water displacement. All lungs were then dehydrated, cleared in toluene and embedded in paraffin. Tissue sections (7 μm) were pre-treated with 3% hydrogen peroxide to inhibit the activity of the endogenous peroxidases. For antigen retrieval, the sections were heated in a microwave for 20 min in citrate buffer 0.01 M, pH 6.0, and allowed to cool slowly to room temperature. The slides were then incubated with 3% bovine serum albumin for 30 min at room temperature to block non-specific antibody binding, and then exposed to the following primary antibodies: prediluted rabbit polyclonal to myeloperoxidase (undiluted) (Abcam, Cambridge, UK), rat monoclonal anti-mouse MAC-3 (1:20) (BD Pharmingen, Buccinasco, Italy), rat monoclonal anti-mouse CD45/B220 (1:20) (BD Pharmingen, Buccinasco, Italy), rat monoclonal anti-mouse to CD4 (1:1000) (Abcam, Cambridge, UK), overnight at 4°C.

For myeloperoxidase detection, the tissue sections were rinsed and incubated with sheep anti-rabbit IgG (1:100) (Sigma Immuno Chemicals) for 30 min. at room temperature. The staining was revealed by adding peroxidase-antiperoxidase complex (1:200) (Sigma Immuno Chemicals) prepared from rabbit serum.

For MAC-3, CD4 and CD45R/B220 detection, the sections were rinsed and incubated with goat polyclonal anti-rat biotinylated IgG (1:100) (Abcam, Cambridge, UK) for 30 min. at room temperature. The staining was revealed by adding Streptavidin-horseradish peroxidase (BD Pharmingen, Buccinasco, Italy).

Detection was accomplished by incubation in diaminobenzidine (DAB) freshly dissolved in 0.03% H_2_O_2 _in 50 mM Tris/HCl, pH 7.6. As negative controls for the immunostaining, the primary Ab was replaced by nonimmunized rabbit or rat serum.

The M.O.M. immunodetection kit (Vector Laboratories, Burlinghame, CA) was used for both immunodection of mouse monoclonal to CD8-α (1:100) (Santa Cruz Biotecnology Inc, Europe) and to fascin (1:200) (Abcam, Cambridge, UK), a protein highly restricted to dendritic cells deriving from the different dendritic cell subsets [10]. Fascin is specifically expressed only in mature but not in immature dendritic cells [11] and its expression is a prerequisite for full T-cells activation [12].

The Vector M.O.M. immunodetection kit is designed specifically to localize mouse primary monoclonal and polyclonal antibodies on mouse tissues by using a novel blocking agent and reducing the undesired background staining. Immunostaining was revealed by using the M.O.M. detection kit with DAB substrate.

The volume fractions of the immunopositive cells were determined by point counting using a grid with 48 points, a 20× objective and a computer screen for a final magnification of 580×. Twenty fields were examined for each pair of lungs for a total of 960 points. The number of points that fell on stained cells was divided by the total number of points on the lung section. This number was then multiplied by the volume of the lung corrected by the weight of the mouse to give the total volume of the specifically stained cells in the lung [13].

The number of animals (N) was 5 in all groups except in the group "smoke: macrophages" where N = 7; "smoke+R1: macrophages" where N = 6; and "smoke+R5: macrophages" where N = 7.

The significance of the differences was calculated using one-way analysis of variance (ANOVA). A p-value of < 0.05 was considered significant.

## Results

Following chronic smoke exposure, the inflammatory cells of both the innate immune system and of the adaptive immune system were significantly increased in the lung tissue when compared to air exposure.

**Figure 1:**
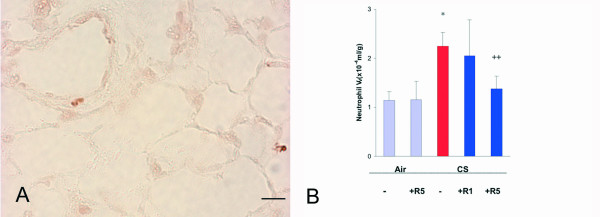
**A**. Immunohistochemical reaction for neutrophil myeloperoxidase on a lung of a C57Bl/6J mouse after a 6 month cigarette smoke exposure. **B**. Neutrophil volume density in the lung of C57BL76J mice exposed either to room air or to cigarette smoke for 6 months and treated or not with roflumilast at 2 doses. Air = air exposure; CS = cigarette smoke exposure; R1 = treated with roflumilast at the dose 1 mg/kg po; R5 = treated with roflumilast at the dose 5 mg/kg po; N = 5 in all groups; * = p < 0.01 versus air exposed, ++ = < 0.01 versus smoke exposed. Scale Bar = 40 μm.

The neutrophils, which could be observed mainly peribronchially but also in the lung parenchyma (Fig. 1A), were increased by 97% (p < 0.01, Fig. 1B). Roflumilast, at the dose 1 mg/kg, did not significantly affect this increase, while at the dose 5 mg/kg, prevented the increase in neutrophil V_V _by 78% (p < 0.01) (Fig. 1B).

**Figure 2:**
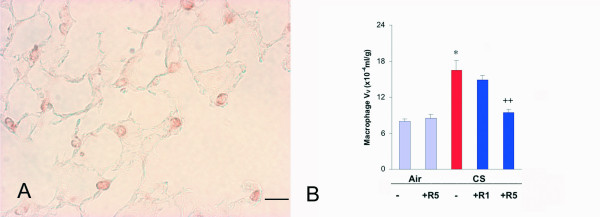
**A**. Immunohistochemical reaction for macrophage Mac-3 on lung parenchyma of a C57Bl/6J mouse after a 6 month cigarette smoke exposure. **B**. Macrophage volume density in the lung of C57BL76J mice exposed either to room air or to cigarette smoke for 6 months and treated or not with roflumilast at 2 doses. Air = air exposure; CS = cigarette smoke exposure; R1 = treated with roflumilast at the dose 1 mg/kg po; R5 = treated with roflumilast at the dose 5 mg/kg po; N = 5 in all groups except "CS -" where N = 7, "CS +R1"where N = 6 and "CS +R5" where N = 7; * = p < 0.01 versus air exposed, ++ = < 0.01 versus smoke exposed. Scale Bar = 40 μm.

The macrophages, which were observed throughout the lung parenchyma (Fig. 2A) were increased by 107% (p < 0.01) in comparison to air exposure (Fig. 2B). Roflumilast, at the dose 1 mg/kg, did not significantly affect (by 19%) the cigarette smoke-induced increase in macrophage V_V_, but at the dose 5 mg/kg suppressed the increase in macrophage V_V _by 82% (p < 0.01) (Fig. 2B). This effect is slightly more potent of what previously reported [6].

**Figure 3:**
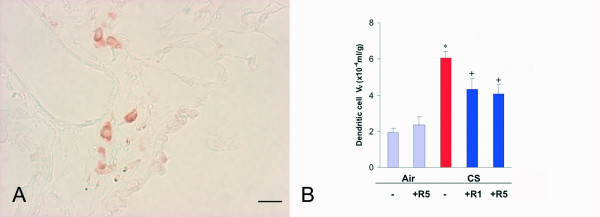
**A**. Immunohistochemical reaction for dendritic cell fascin observed perivascularly on a lung of a C57Bl/6J mouse after a 6 month cigarette smoke exposure. **B**. Dendritic cell volume density in the lung of C57BL76J mice exposed either to room air or to cigarette smoke for 6 months and treated or not with roflumilast at 2 doses. Air = air exposure; CS = cigarette smoke exposure; R1 = treated with roflumilast at the dose 1 mg/kg po; R5 = treated with roflumilast at the dose 5 mg/kg po; N = 5 in all groups except "CS +R1"where due to technical problems N = 3; * = p < 0.01 versus air exposed, + = < 0.05 versus smoke exposed. Scale Bar = 40 μm.

The dendritic cells expressing fascin were increased by 217% (p < 0.01) (Fig. 3B). These cells were found perivascularly (Fig. 3A) in the airway walls and in the parenchyma, as well as organized in lymphoid follicles. Roflumilast, at the dose 1 mg/kg reduced by 42% (p < 0.05) (Fig. 3B) the increase in V_V _of mature dendritic cells with the potential ability to activate T-cell proliferation. A similar effect was obtained with the dose 5 mg/kg (-48%, p < 0.05) (Fig. 3B).

**Figure 4:**
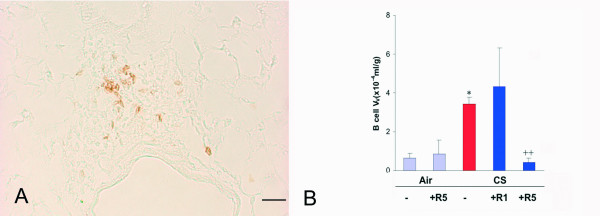
**A**. Immunohistochemical reaction for B-lymphocyte CD45/B220 organized in a lymphoid follicle on a lung of a C57Bl/6J mouse after a 6 month cigarette smoke exposure. **B**. B-cell volume density in the lung of C57BL76J mice exposed either to room air or to cigarette smoke for 6 months and treated or not with roflumilast at 2 doses. Air = air exposure; CS = cigarette smoke exposure; R1 = treated with roflumilast at the dose 1 mg/kg po; R5 = treated with roflumilast at the dose 5 mg/kg po; N = 5 in all groups. * = p < 0.01 versus air exposed, ++ = < 0.01 versus smoke exposed. Scale Bar = 80 μm.

B-lymphocytes, which were exclusively found organized in the lymphoid follicles observed both peribronchially (Fig. 4A) and in the parenchyma, were increased by 436% (p < 0.01) (Fig. 4B). The low dose of roflumilast potentiated, not significantly, by 26% the increase in B cell V_V_. This increase was due to a very high value observed in one lung of this group and this is reflected by the unusually high SEM of this group. Roflumilast, at the dose 5 mg/kg, prevented the increase in B-lymphocyte V_V _by 100% (p < 0.01) (Fig. 4B).

**Figure 5:**
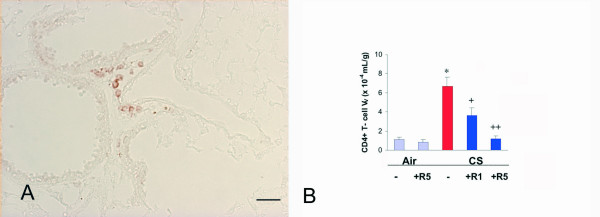
**A**. Immunohistochemical reaction for CD4+ T-lymphocyte CD4 seen perivascularly on a lung of a C57Bl/6J mouse after a 6 month cigarette smoke exposure. × 250. **B**. CD4+ T-cell volume density in the lung of C57BL76J mice exposed either to room air or to cigarette smoke for 6 months and treated or not with roflumilast at 2 doses. Air = air exposure; CS = cigarette smoke exposure; R1 = treated with roflumilast at the dose 1 mg/kg po; R5 = treated with roflumilast at the dose 5 mg/kg po; N = 5 in all groups. * = p < 0.01 versus air exposed, + = < 0.05 versus smoke exposed, ++ = < 0.01 versus smoke exposed. Scale Bar = 80 μm.

The CD4+ T-lymphocytes, which were found perivascularly (Fig. 5A), in the airways and in the lymphoid follicles were increased in the lungs of the mice exposed to cigarette smoke by 524% (p < 0.01) (Fig. 5B). The low dose of roflumilast reduced the increase in CD4+ cell V_V _by 55% (p < 0.05) and the high dose by 98% (p < 0.01) (Fig. 5B).

**Figure 6:**
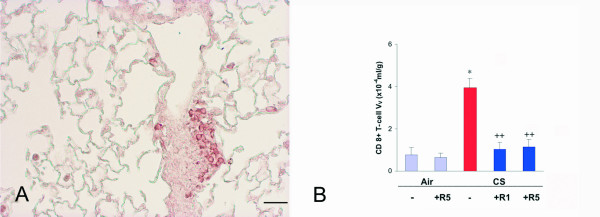
**A**. Immunohistochemical reaction for CD8+ T-lymphocyte CD8-α seen organized in a lymphoid follicle on a lung of a C57Bl/6J mouse after a 6 month cigarette smoke exposure. **B**. CD8+ T-cell volume density in the lung of C57BL76J mice exposed either to room air or to cigarette smoke for 6 months and treated or not with roflumilast at 2 doses. Air = air exposure; CS = cigarette smoke exposure; R1 = treated with roflumilast at the dose 1 mg/kg po; R5 = treated with roflumilast at the dose 5 mg/kg po; N = 5 in all groups. * = p < 0.01 versus air exposed, ++ = < 0.01 versus smoke exposed. Scale Bar = 80 μm.

Following chronic exposure to cigarette smoke CD8+ T-lymphocytes were visualized perivascularly, peribronchially, and in lymphoid follicles (Fig. 6A) and were increased by 417% (p < 0.01) (Fig. 6B). Both doses of roflumilast showed a similar inhibition of the influx of these cells: 91% at 1 mg/kg and 88% at 5 mg/kg, respectively (both p < 0.001) (Fig. 6B).

## Discussion

The main points of this study are: (*i*) the effect of chronic smoke exposure on the recruitment of inflammatory cells of both the innate and adaptive immune system and (*ii*) the effect of the two doses of roflumilast on this recruitment.

With regard to inflammatory cells of the innate immune system, cigarette smoke exposure resulted in an increase of approximately 100% of both neutrophil and macrophage V_V_. In particular, neutrophil V_V _was increased by 97% after 7 months cigarette smoke exposure. This result is consistent with the data of a recent report where neutrophils, also assessed morphometrically, were increased by 155% in the lung of C57 Bl/6J mice exposed for one year to cigarette smoke [14]. A pronounced increase of neutrophils was found in bronchoalveolar lavage fluids (BALFs) of mice with the same background and after exposure to cigarette smoke for 6 months [15,16].

In COPD in man, an increased number of neutrophils is recovered from sputum and BALFs however, there are relatively small increases of neutrophils in the airways or parenchyma [17]. The lack of a significant increase in neutrophils in the lung parenchyma has been attributed to the rapid transit that these cells make through the airways and the lung parenchyma [18]. In our opinion, the discrepancy between animal and human data may be explained by the different methodology used for performing bronchoalveolar lavage that is known to be representative in humans of a small region of the lung (segmental lavage).

In the present study, cigarette smoke exposure resulted in an increase in lung macrophage V_V _by 107%. Similarly, in a recent study macrophages were increased by 81% in mice exposed to cigarette smoke for one year as compared to air exposed animals [13]. Further, an investigation of the time course of the increase of inflammatory cells following cigarette smoke exposure revealed a progressive biphasic increase in BALF macrophages. There was a slight but significant increase after 7 days, and a marked increase after 6 months. In the lung, the number of macrophages increased significantly after 12 weeks and remained elevated up to 6 months. [15]. Similarly, in patients with COPD, there is a marked increase in the number of macrophages both in BALF and in the lung parenchyma [19].

In the present study activated dendritic cells, B-lymphocytes and CD4+ and CD8+ T-lymphocytes were also markedly increased after cigarette smoke. The results of the dendritic cells are supported by recent data obtained in the mouse showing a strong increase of dendritic cells in the lung in response to cigarette smoke [15,20]. The dendritic cells are antigen presenting cells and the most likely explanation for the increased number of these cells could be their recruitment as a response either to smoke inhalation or to tissue damage. Dendritic cells would then take up, process and present antigenic substances contained either in cigarette smoke or extracellular matrix degradation products to T-cells. Additionally, dendritic cells are not only antigen presenting cells but also following cigarette exposure they produce MMP-12 thus, having the potential for a direct damaging effect [21].

The present results regarding the T-cells are consistent with the data of a recent work where the total number of T-lymphocytes was assessed in the lung of mice following cigarette smoke exposure. At 6 months, total number of CD4+ T-lymphocytes was increased 2-fold while CD8+ T-lymphocytes increased by 43%. Additionally, the number of activated CD4+ and CD8+ T-cells was also found to be increased [15]. In an attempt to elucidate the role for CD8+ cells in emphysema, CD8+ T cell-deficient (CD8-/-) mice were chronically exposed to cigarette smoke. In contrast to wild-type mice that displayed macrophage, lymphocyte, and neutrophil recruitment followed by emphysema, CD8-/- mice had a blunted inflammatory response and did not develop emphysema. The hypothesis was put forward whereby the CD8+ T-cell product, IFN-gamma-inducible protein-10, would induce production of MMP-12 causing lung destruction and generating chemotactic factors [22].

In the present study B-lymphocytes, which were found in peribronchial and parenchymal lymphoid follicles, were increased by more than 4-fold. Additionally as mentioned above, mature dendritic cells, CD4+ and CD8+ T-lymphocytes were also seen in the lymphoid follicles. Although CD4 and CD8a are expressed in some monocyte/macrophages or in some KN and dendritic cells, our findings are consistent with recent data reported in the mouse after chronic smoke exposure [23]. The formation of lymphoid follicles defines the appearance of an adaptive immune response [24], and the colocalisation of lymphocytes and mature dendritic cells in one site suggests that these cells are functionally communicating [25].

The question that arises is what is the potential role of the B-cells in the development of emphysema. At present, is unclear against which antigen this B-cell proliferation may be directed. The hypothesis of an antigen of microbial nature has been put forward in man [9]. Additionally, as mentioned above, there are at least two alternative potential sources that should be considered, cigarette smoke components and degradation products of extracellular matrix [7]. Another hypothesis suggests that cigarette smoke, via an oxidative challenge, could modify lung proteins in such a way to make them immunogenic [26].

In COPD patients all these cells of the adaptive immune system are prominent [27-29] however, their specific role in the development of the disease is still a matter of discussion. Similarly, the role of the innate immunity in the development of cigarette smoke induced emphysema has not been clarified.

In the present study roflumilast, at the dose of 5 mg/kg provided a marked and significant protection against the influx into the lungs of the inflammatory cells of both the innate and adaptive immune system and this dose of roflumilast has been previously reported to completely prevent the development of emphysema [6]. Thus, the present results would support a role for all these inflammatory cells in the development of emphysema and suggest that inhibition of their recruitment may prevent the development of the disease.

However, the low dose of roflumilast had no effect on neutrophil and macrophage V_V _but prevented the increase of dendritic cells with the potential to activate T-cell proliferation by 42%, that of CD+4 positive cells by 55% and almost completely (91%) blocked that of CD8+ positive cells. This low dose of roflumilast did not have any effect on the development of emphysema [6].

All these data taken together could be interpreted to signify that the inflammatory cells of the innate immune system are likely to play a major role in the development of emphysema. This, since their influx is associated with the presence of this disease and conversely when their recruitment is prevented there is no emphysema (as it is the case with the dose 5 mg/kg of roflumilast). On the other hand, the inflammatory cells of the adaptive immune system probably do not play a major role since emphysema can fully develop even when the activation of dendritic cells, and the number of CD4+ and CD8+ positive cells is significantly reduced or blocked (as with the dose 1 mg/kg roflumilast).

This conclusion is consistent with the results of a previous study performed in SCID mice chronically exposed to cigarette smoke. These mice lack functional B- and T-cells and peribronchial lymphoid follicles. In these mice cigarette smoke induced a progressive increase of neutrophils and macrophages in BALF, and induced significant emphysema. [30].

In a recent work Robbins and co-workers specifically investigated the impact of chronic tobacco smoke exposure on respiratory immune defence mechanisms [31]. C57Bl/6J mice were chronically exposed to cigarette smoke and emphysematous lesions were observed after 6 months and were even more pronounced by 10 months. Cigarette smoke exposure reduced the number and the maturation of dendritic cells in the lung by altering their costimulatory molecule expression profile without affecting the CD4+ and CD8+ T-cell compartments. The differences in dendritic as well as in CD4+ and CD8+ T-cell profiles observed in different studies dealing with smoking mice may be accounted to the different methodological procedures (i.e. manner of smoking, time of exposure, number or type of cigarette) in use in different labs. All these data taken together strongly suggest that the cells of the adaptive immune system do not play a major role in the development of emphysema in smoking mice. However, we cannot exclude at the present time a role for adaptive immune responses in the progression of emphysematous lesions induced by cigarette smoking.

## Conclusion

In conclusion the results of the present study coupled with the data of the literature indicate: (*i*) chronic exposure to cigarette smoke in mice results in a significant recruitment into the lung of inflammatory cells of both the innate and adaptive immune system, as assessed by morphometry; (*ii*) roflumilast at the higher dose exerts a protective effect against the recruitment of all these cells and at the lower dose against the recruitment of dendritic cells and T-lymphocytes; (*iii*) these findings underline the role of innate immunity in the development of pulmonary emphysema and (*iiii*) support previous results indicating that the inflammatory cells of the adaptive immune system do not play a central role in the development of cigarette smoke induced emphysema in mice.

## Competing interests

PAM, GL, BL, ML and GDC, have no financial competing interests associated with this study. RB is employed by Nycomed GmbH.

## Authors' contributions

PAM contributed to the morphometrical analysis and wrote the draft of the manuscript. PAM, RB and GL participated in the design of the study, analysed the results and contributed to the final version of the manuscript. ML coordinated the animal experimentation. ML, BL, and GDC performed the histochemical reactions and participated to the morphometrical analysis. All authors read and approved the final manuscript.

## Pre-publication history

The pre-publication history for this paper can be accessed here:


